# Recent Advances in Multinuclear NMR Spectroscopy for Chiral Recognition of Organic Compounds

**DOI:** 10.3390/molecules22020247

**Published:** 2017-02-07

**Authors:** Márcio S. Silva

**Affiliations:** Centro de Ciências Naturais e Humanas—CCNH—Universidade Federal do ABC—UFABC, Av. Dos Estados 5001, 09210-180 Santo André –SP, Brazil; s.marcio@ufabc.edu.br; Tel.: +55-11-4996-8358

**Keywords:** chiral recognition, NMR spectroscopy, chirality, multinuclear, enantiopurity, enantiomeric excess, stereochemistry, absolute configuration

## Abstract

Nuclear magnetic resonance (NMR) is a powerful tool for the elucidation of chemical structure and chiral recognition. In the last decade, the number of probes, media, and experiments to analyze chiral environments has rapidly increased. The evaluation of chiral molecules and systems has become a routine task in almost all NMR laboratories, allowing for the determination of molecular connectivities and the construction of spatial relationships. Among the features that improve the chiral recognition abilities by NMR is the application of different nuclei. The simplicity of the multinuclear NMR spectra relative to ^1^H, the minimal influence of the experimental conditions, and the larger shift dispersion make these nuclei especially suitable for NMR analysis. Herein, the recent advances in multinuclear (^19^F, ^31^P, ^13^C, and ^77^Se) NMR spectroscopy for chiral recognition of organic compounds are presented. The review describes new chiral derivatizing agents and chiral solvating agents used for stereodiscrimination and the assignment of the absolute configuration of small organic compounds.

## 1. Introduction

Stereoisomers are compounds with the same molecular formula, possessing identical bond connectivity but different orientations of their atoms in space [1]. Enantiomers are stereoisomers that are mirror images of each other, but at the same time are not superimposable. Chirality is important in chemical, physical, pharmaceutical, and biological systems, inspiring new biomimicry-based innovations [2,3]. Moreover, the need to discriminate between enantiomers and quantify enantiomeric excess (ee) is of extreme importance in the pharmaceutical industry and for asymmetric synthesis [4,5,6].

Nowadays, the use of chromatography separation of enantiomers on chiral stationary phases is still the approach most often applied in modern chemical research [7,8]. However, the search for new chiral discriminating procedures that allow for quick analysis, high resolution, and utility for many non-volatile or thermally unstable compounds is increasing [9,10,11,12]. Among the several stereodiscrimination methods, including X-ray, circular dichroism, fluorescence spectroscopy, and electrophoresis, nuclear magnetic resonance (NMR) spectroscopy continues to be a useful tool for determining the enantiomeric purity and assigning the absolute configuration of chiral molecules [13,14,15,16,17,18,19,20,21].

NMR active nuclei are isochronous in an achiral medium and do not permit their discrimination, but in a chiral environment these nuclei are anisochronous and chiral discrimination is possible. Therefore, to perform the enantiopurity analysis, a chiral derivatization or solvating agent is essential to produce a nonequivalent diastereomeric mixture and relevant differences in the NMR spectra. Chiral derivatizing agents form a covalent bond with a reactive moiety of the substrate and chiral solvating agents associate with the substrate through non-covalent interactions, such as dipole–dipole and ion pairing. In this context, strategies based on different intermolecular reactivities, interactions, and packing orders for a pair of enantiomers are in constant development.

Among the active NMR nuclei, ^1^H is the most important. The characteristics of ^1^H, such as its natural abundance (99.98%) and its high sensitivity to environmental modifications, make it immensely versatile in NMR chiral analysis [22]. Nevertheless, ^1^H-NMR spectroscopy poses some limitations. The ^1^H-NMR spectra for chiral analysis are severely hampered due to the numerous scalar couplings, and the overlap combined with broad and featureless spectra leads to enormous difficulties in ^1^H-NMR analysis, even for small molecules. Consequently, the comparison of the enantiomers using NMR spectra and the assignment of absolute configuration can be unclear. The application of different NMR nuclei, mainly ^19^F and ^31^P, overcomes these limitations. The simplicity of the multinuclear NMR spectra relative to the ^1^H and the larger shift dispersion make these nuclei especially suitable for analysis.

In this review, new chiral derivatization agents (CDAs), chiral solvating agents (CSAs), and modern methods for stereodiscrimination and assignment of the absolute configuration of organic compounds by ^19^F-, ^31^P-, ^13^C-, and ^77^Se-NMR spectroscopy are described. The focus is on articles from 2007 to the present date. Furthermore, the ^2^H nucleus is not described because of the necessity of discussing the physical bases in order to understand the quadrupolar electric moment and residual dipolar coupling contents [23].

## 2. ^19^F-NMR Chiral Recognition

^19^F is one of the most useful nuclei for NMR studies [24]. The fluorine element has polar and steric properties as a substituent and the effects that fluorinated groups can have on the physical and chemical properties of molecules have increased the number of new methods for incorporating fluorine into target compounds. Furthermore, ^19^F-NMR spectroscopy has proved to be a valuable tool to study the structure and function of nucleic acids by incorporating fluorine labels into DNA and RNA [25].

Since the ^19^F-NMR frequency and sensitivity are similar to the proton (the fluorine spectra is at 282 MHz and the proton spectrum is at 300 MHz), it is convenient to transition from proton to fluorine NMR experiments.

**Scheme 1:**
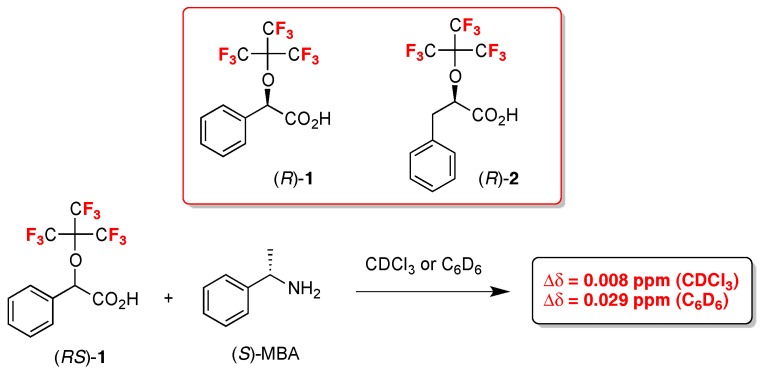
α-(nonafluoro-*tert*-butoxy)carboxylic acid as an ^19^F {^1^H} NMR CDA.

A wide variety of fluorine-containing reagents have been developed for NMR chiral discrimination [26,27]. In 2015, Nemes et al. described the chiral recognition studies of α-(nonafluoro-*tert*-butoxy)carboxylic acid by ^19^F-NMR spectroscopy [28]. Carboxylic acids **1** and **2** were synthesized to be enantiomerically enriched in order to obtain a CDA (Scheme 1). The synthesis of racemic carboxylic acids **1** and **2** was carried out in two steps. Chiral recognition studies were performed with (*S*)-phenylethylamine. In the NMR measurements, C_6_D_6_ solvent was more favorable than CDCl_3_ in the differences (Δδ) of diastereomeric signals. The benefits of these CDAs include the presence of an acidic functional group for a strong interaction, a bulky hydrophobic group OC(CF_3_)_3_ and an aromatic ring for a π–π interaction. Additionally, the ^19^F-NMR spectra have only one strong singlet to nine chemically equivalent fluorine atoms.

**Scheme 2:**
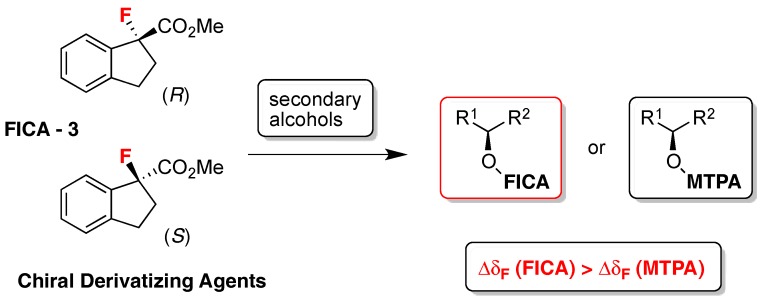
1-fluoroindan-1-carboxylic acid **3** (FICA) as a chiral derivatizing agent for ^19^F {^1^H} NMR spectroscopy.

Another fluorine-containing CDA that surpasses the capabilities of Mosher’s acid (MTPA) was prepared by Takahashi et al. (Scheme 2) [29]. To obtain the chiral compounds (*R*)-**3** and (*S*)-**3**, high-performance liquid chromatography (HPLC) was carried out. The efficiency of the ^19^F-NMR nucleus was evaluated with secondary alcohols. The absolute configuration of chiral FICA **3** was determined by reaction with (*R*)-phenylethylamine following by single-crystal X-ray crystallographic analysis. The magnitude of the chiral discrimination of the FICA esters was larger than that of the MTPA esters (Scheme 2). Moreover, the assignment of the absolute configuration of the alcohols was easily performed by ^19^F-NMR spectroscopy.

**Scheme 3:**
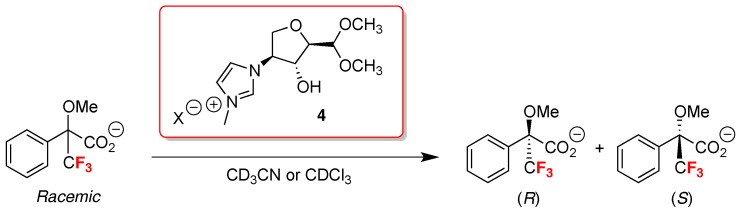
Chiral ionic liquid **4** as a chiral solvating agent for ^19^F {^1^H} NMR spectroscopy.

A chiral ionic liquid **4** was employed by Reddy et al. for chiral recognition of racemic Mosher’s acid salt using ^19^F-NMR spectroscopy [30]. Ionic liquids (ILs) have received considerable attention as substitutes for volatile organic solvents due to their remarkable properties, such as non-flammable, non-volatile and recyclable. Thus, a chiral ionic liquid containing D-xylose was synthesized in seven steps with a good overall yield (56%). The D-xylose is a cheap and readily available starting material. The chiral recognition study was performed with the racemic Mosher’s acid silver salt in CD_3_CN (Scheme 3). The concentration was varied to observe the signals splitting and line shape of ^19^F-NMR. The effectiveness of the chiral ionic liquid **4** was also tested by using a non-racemic Mosher’s acid salt. The enantiomeric excess (ee) of this non-racemic salt was determined by ^19^F-NMR. Moreover, the splitting pattern of the chiral ionic liquid **4** was evaluated by ^1^H- and ^13^C-NMR spectroscopy.

**Scheme 4:**
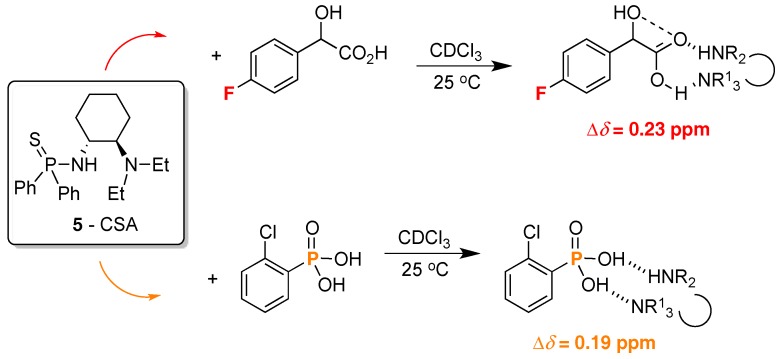
Chiral thiophosphoramide **6** as a CSA for a variety of carboxylic acids by ^19^F {^1^H} and ^31^P {^1^H} NMR spectroscopy.

Huang et al. described a CSA for a variety of acids using chiral thiophosphoramide **5** derived from (1*R*,2*S*)-1,2-diaminocyclohexane (Scheme 4) [31]. The interaction via ion pairing and hydrogen bonding provides a large split in values between the enantiomers, especially when the ^19^F-NMR was evaluated. The authors completed the chiral recognition study with phosphonic acids by ^31^P-NMR spectroscopy (Scheme 4).

**Scheme 5:**
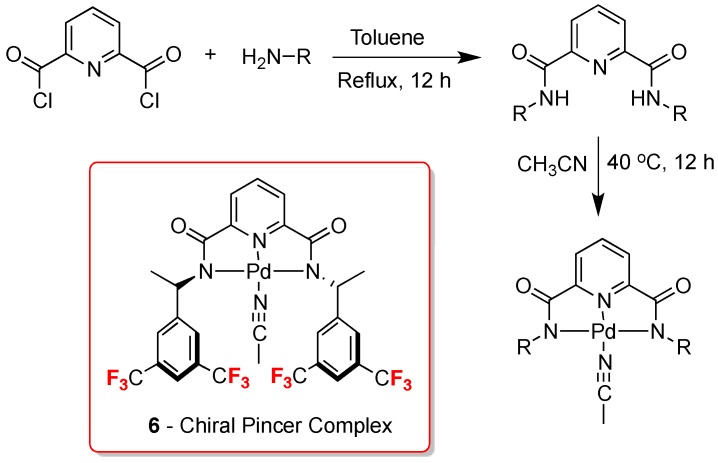
Chiral amide-based palladium complex **6** as a chiral derivatizing agent for ^19^F {^1^H} NMR spectroscopy.

Swager and Zhao prepared amide-based palladium pincer complex **6** as a scaffold to examine the chiral discrimination ability of amines (Scheme 5) [32]. Based on well-known coordination chemistry, the complex was easily synthesized in two steps. The chiral sensor site that undergoes facile ligand exchange is flanked by fluorine pendant groups that are sensitive to enantiomers. The observation of split signals at precise chemical shifts that are not concentration-dependent indicated the formation of static complexes on the NMR time scale. Moreover, the accuracy of the method was evaluated by measuring the enantiomeric excess of the amines with different ratios.

**Scheme 6:**
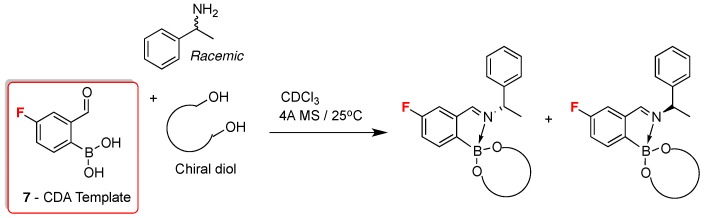
Three-component coupling reaction for enatiopurity determination of chiral diols by ^19^F {^1^H} NMR spectroscopy.

A practical and simple derivatization protocol for determining the enantiopurity of chiral diols by ^19^F-NMR spectroscopy was developed by James et al. [33]. The experimental procedure consisted of mixing an equimolecular amount of 4-fluoro-2-formylphenyl boronic acid **7**, phenylethylamine, and the chiral diol in CDCl_3_ at 25 °C (Scheme 6). The focus of the method was to synthesize the fluorine compound **7** as a new bifunctional template for carrying out the enantiopurity analysis of chiral diols.

**Scheme 7:**
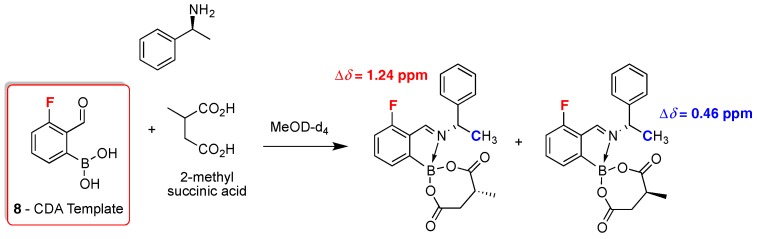
Three-component coupling reaction for enatiopurity determination of diacids by ^19^F {^1^H} and ^13^C {^1^H} NMR spectroscopy.

A closely related approach was used by Chaudhari and Suryaprakash to perform the discrimination of chiral diacids and chiral *alpha* methyl amines [34]. The three-component chiral derivatization protocol has been developed for ^19^F- and ^13^C-NMR. These methodologies are based on a mixture of diastereomeric iminoboronate esters without any racemization or kinetic resolution. The Suryaprakash method was the first procedure for determining the enantiopurity of chiral diacids. The general protocol involves the reaction of an equimolecular amount of diacids with 3-fluoro-2-formylboronic acid **8** and an enantiopure (*R*)-methylethylamine in methanol-*d*_4_ (Scheme 7). The baseline separation of ^19^F and ^13^C was 1.24 ppm and 0.46 ppm, respectively, for the *rac*-2-methylsuccinic acid (Scheme 7).

There is great potential for the use of the boronic acid derivatives mentioned above and other related procedures [35,36,37] in the chiral recognition of organic and water soluble compounds due to their use as new probes, mainly in biologically relevant species [38]. The boronic acids have Lewis acid characteristics and react spontaneously and reversibly with diol compounds. Furthermore, boron chemistry is closely related to the living systems. These characteristics, in addition to the usefulness of multinuclear NMR spectroscopy, represent valuable methodologies to discriminate chiral organic compounds.

**Scheme 8:**
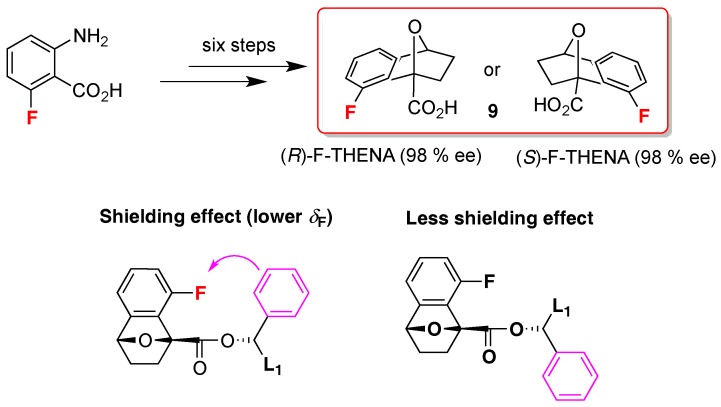
Assignment of absolute configuration of secondary amines and alcohols employing the F-THENA-Cl CDA by ^19^F {^1^H} NMR spectroscopy.

Another chiral fluorine-containing carboxylic acid, F-THENA **9**, was recently prepared by Dolsophon et al. The rigid anisotropic structure of F-THENA **9** is useful for assigning the absolute configuration of secondary alcohols and amines by ^19^F-NMR spectroscopy [39]. The CDA was synthesized from a commercially available starting material in six steps (Scheme 8). To establish the absolute configuration, X-ray analysis was performed. Derivatizations of the (*S*) and (*R*) F-THENA **9** acids with oxalyl chloride in the presence of a catalytic amount of dimethylformamide allowed for the formation of (*S*) and (*R*) F-THENA-Cl. In ^19^F-NMR, a positive shielding effect occurs from the alcohol moiety when an aromatic group is located on the lower side of the F-THENA plane while a negative value is obtained when an aromatic group is located on the upper side of the F-THENA plane (Scheme 8). The fluorine-containing CDA provides a self-validating system, which reduces the risks of incorrect assignment. The NMR results were strongest when both configuration assignments, from ^19^F- and ^1^H-NMR, were identical.

**Scheme 9:**
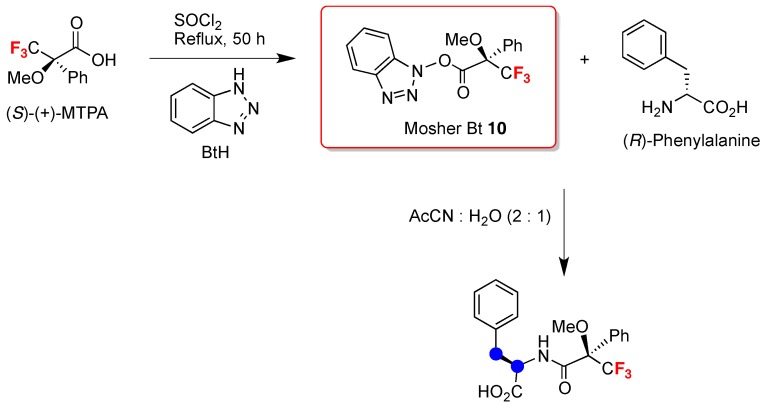
Assignment of the absolute configuration of amino acids by ^19^F {^1^H} and ^13^C {^1^H} NMR spectroscopy.

An alternative approach to assign the absolute configuration of amino acids using the Mosher’s acid was developed by Katritzky et al [40]. *N*-acylbenzotriazoles are stable and crystalline derivatives of carboxylic acids and have advantages mainly when compared to acid chlorides that are unstable, moisture sensitive, difficult to prepare, and need to be stored in a deep freeze. Thus, the authors synthesized the Mosher-Bt **10** reagent (Scheme 9) from chiral and racemic Mosher’s acid to employ as a new and stable CDA for amino acids and peptides. The carboxylic acid derivative **10** was easily prepared with thionyl chloride and 1*H*-benzotriazole (BtH) and their reactions were studied with representative water-soluble chiral amino acids and peptides in acetonitrile-water medium (Scheme 9). The assignment of the absolute configuration of (*R*)-phenylalanine using the Mosher-Bt **10** reagent was performed using ^1^H-, ^19^F-, and ^13^C-NMR spectroscopy. To demonstrate that racemization does not occur during the reaction, Mosher amides were subjected to HPLC analysis. Recently, to understand the conformational features of amide scaffolds in the assignment of the absolute configuration, Ichikawa et al. studied the characteristic conformation of Mosher’s acid amide, which was elucidated using the Cambridge Structural Database [41]. Amides are important for biological systems and are especially important in the pharmaceutical industry since a huge number of molecular drug candidates have nitrogen atoms [42,43]. Furthermore, chiral amines are also used as the starting material in the formation of new amino acids and peptide bonds.

**Scheme 10:**
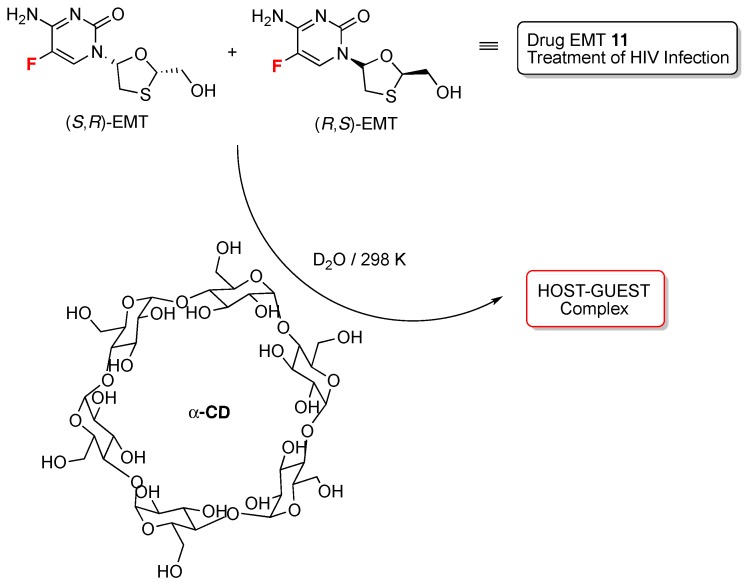
Chiral discrimination of emtricitabine **11** by cyclodextrin host–guest complexes using ^19^F {^1^H} NMR spectroscopy.

Cyclodextrins (CDs) were employed as a chiral recognition agent for enantiodiscrimination of emtricitabine enantiomers **11**, a novel nucleoside reverse transcriptase inhibitors for treatment of HIV infection in adults and children, by ^19^F-NMR spectroscopy [44]. In this protocol, developed by Rao et al., the influence of the CD cavity sizes (α, β, and γ), temperature and concentration were studied. The method is based on intermolecular interaction between emtricitabine enantiomers **11** with cyclodextrins in D_2_O solution (Scheme 10). The hydrophobic nature of such cavities provides a chiral environment for enantiodiscrimination. The α-CD has shown better enantiodiscrimination results employing low concentrations (0.01 mM) and temperatures (298 K). Moreover, the binding constant (K) was determined using the ^19^F-NMR method developed and a 2D ^1^H ROESY NMR analysis was performed to understand the geometry of association with α-CD.

## 3. ^31^P-NMR Chiral Recognition

Organophosphorus is an old field of organic chemistry. Organophosphorus compounds are essential in all chemical sciences, especially the life sciences, because their structures are a key building block [45,46]. Furthermore, the pharmaceutical and agrochemical industries have prompted studies of phosphorus chemistry [47].

Unlike ^19^F-NMR spectroscopy, the main factors that govern the ^31^P chemical shift values are different due to other bond contributions. In this way, a good knowledge of organometallic chemistry could help with understanding those variations.

**Scheme 11:**
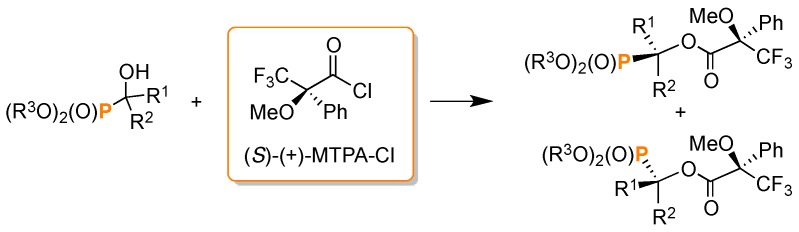
Assigning of the configuration of α-hydroxyphosphonates from derivatization with (*S*)-MTPA-Cl by ^31^P {^1^H} NMR spectroscopy.

^31^P-NMR spectroscopy is highly useful, since compounds from natural resources normally contain this nucleus and ^31^P is more abundant (100%), making it a valuable probe for NMR investigations [48]. Recently, Szyszkowiak and Majewska have reviewed the application of ^31^P-NMR for determination of the absolute configuration of organic compounds [49]. In this review, the model created by Hammersschmidt and Li, in which the shielding of the phosphorus atom by the phenyl group in (*S*)-MTPA ester caused the signal to appear at a higher field (Scheme 11), was explored [50]. This model, based on Mosher’s esters, is currently an important tool for assigning the absolute configuration of many organic functionalities and structures by ^1^H-NMR spectroscopy [51].

**Scheme 12:**
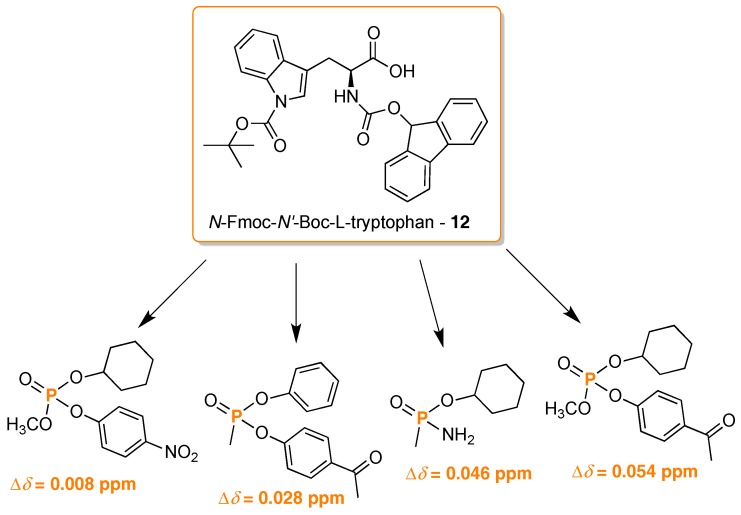
*N*-Fmoc-*N*’-Boc-L-tryptophan **12** as a CSA for chiral discrimination of phosphonates, phosphinates, phosphates, phosphine oxides, and phosphonamidates by ^31^P {^1^H} NMR spectroscopy.

The commercially available amino acid derivatives were employed as a CSA to differentiate enantiomers of chiral phosphonates, phosphinates, phosphates, phosphine oxides, and phosphonamidates by ^31^P-NMR spectroscopy [52]. The method developed by Li and Raushel uses *N*-Fmoc-*N*’-Boc-*L*-tryptophan **12** in CDCl_3_ at 25 °C as a CSA for different phosphorus compounds (Scheme 12). Although there is a lower anisochrony of the phosphorus atoms, the good baseline resolution and absence of overlapping signals provided an efficient and fast chiral discrimination procedure.

**Scheme 13:**
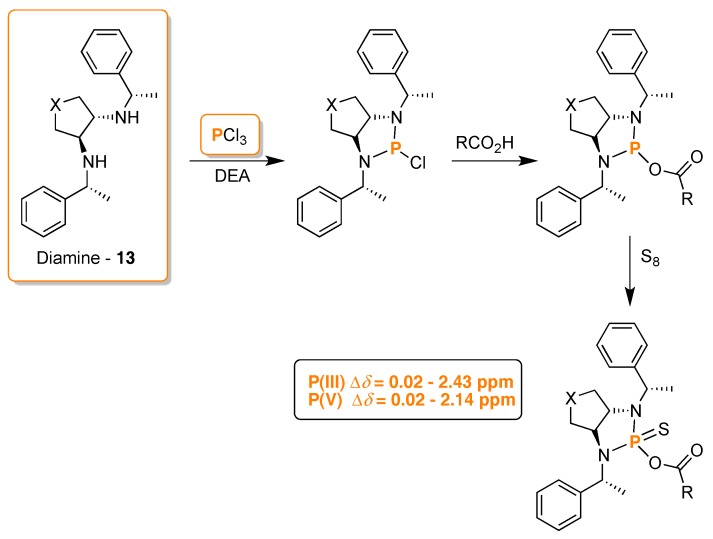
Preparation of organophosphorus CDA for chiral discrimination of carboxylic acids by ^31^P {^1^H} NMR spectroscopy.

Mastranzo et al. demonstrated the use of P(III) and P(V) organophosphorus deriving reagents for chiral discrimination of carboxylic acids by ^31^P-NMR [53]. In this work, the authors described the preparation of *C*_2_ symmetric diamines **13**, which contain the α-phenyl-ethyl group and the cyclohexane skeleton. The discrimination of chiral carboxylic acids was performed in an NMR tube in three reaction steps (Scheme 13). The chemical shift difference between diastereomers varied in the ranges of 0.02–2.43 for P(III) and 0.02–2.14 for P(V).

**Scheme 14:**
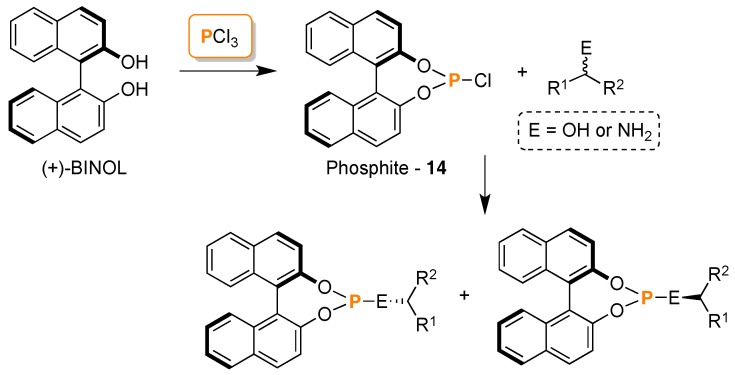
Synthesis of CDA for chiral discrimination of alcohols and amines by ^31^P {^1^H} NMR spectroscopy.

The same approach was employed by Reiner et al. who used ^31^P-NMR to monitor the formation of diastereoisomers by a PCl_3_ reagent [54]. In this procedure, the PCl_3_ reagent reacted with the chiral BINOL to form phosphite derivative **14** and after the alcohol or amine was added to obtain a mixture of diastereomers (Scheme 14). The chiral discrimination was possible by two diastereomeric ^31^P-NMR peaks for both enantiomers of the compounds. The measurements were performed in less than 5 min with only 500 μL of deuterated solvent. Moreover, the authors examined its application to on-line ee determination in combination with standard catalytic protocols.

**Scheme 15:**
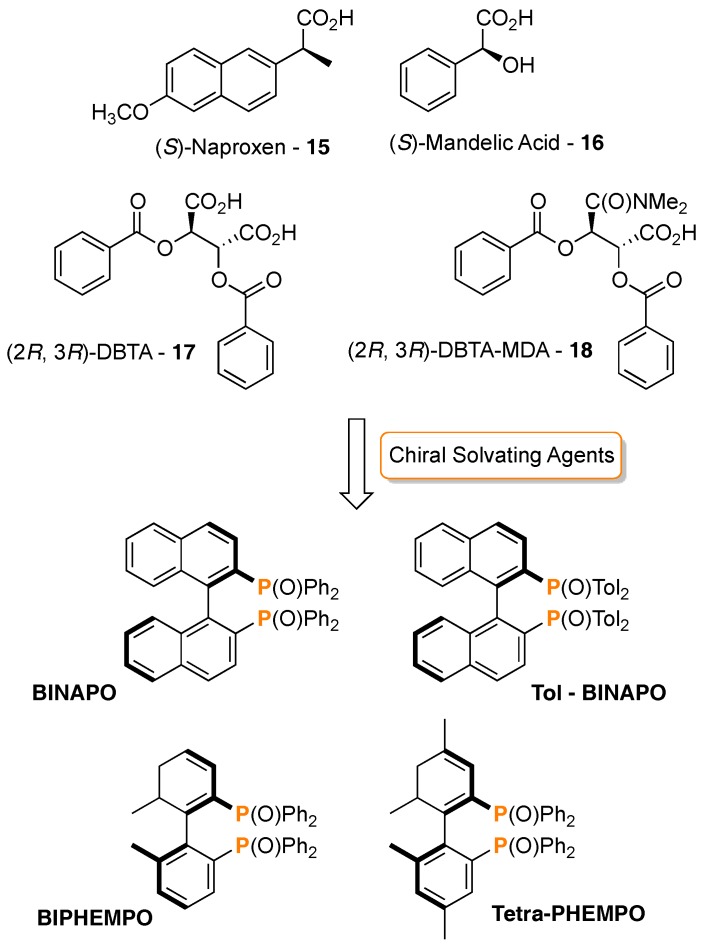
Chiral carboxylic acids used as CSAs for enantiodiscrimination of bis-phosphine dioxides by ^31^P {^1^H} NMR spectroscopy.

For ^31^P-NMR chiral discrimination of atropoisomeric phosphine oxides, Demchuk et al. evaluated different CSAs [55]. The commercially available carboxylic acids **15** and **16**, which have provided good results for NMR chiral discrimination [56,57], and dibenzoyltartaric acids **17** and **18** were applied as CSAs (Scheme 15). The ^31^P-NMR analyses have shown that in most cases those simple chiral acids are very efficient CSAs for the determination of the enantiopurity of atropoisomeric bis-phosphine dioxides. To prove the accuracy of the NMR methodology an electronic circular dichroism spectroscopy was applied. The electronic absorptions and chiroptical data of the investigated compounds corroborate the NMR analyses. Furthermore, computer calculations were carried out to provide details about the stereochemistry of the complexes.

**Scheme 16:**
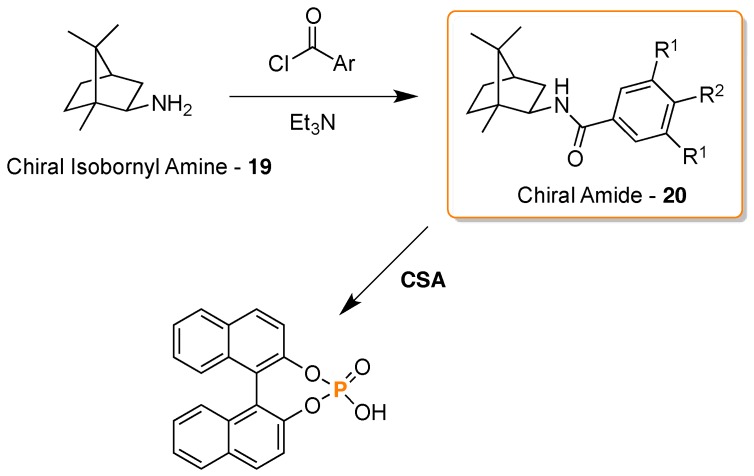
Synthesis of isobornyl derived amides as CSA for enantiodiscrimination of binaphthyl by ^31^P {^1^H} NMR spectroscopy.

**Scheme 17:**
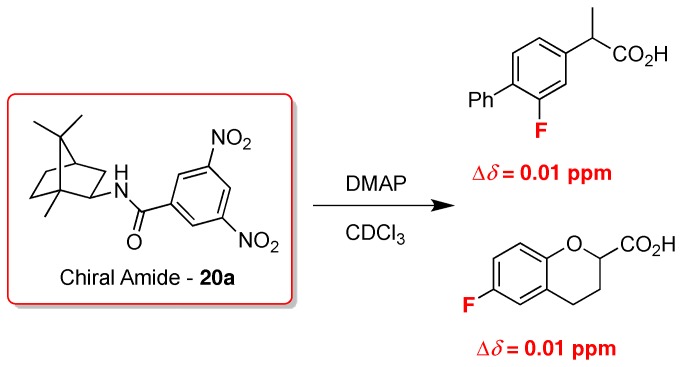
Chiral discrimination of carboxylic acids by ^19^F {^1^H} NMR spectroscopy.

Bedekar et al. synthesized modified amides as NMR solvating agents for the chiral discrimination of 1,1′-binaphthyl and *alpha*-substituted acid enantiomers [58]. In this procedure, the chiral isobornyl amine **19** was employed, which is readily available and can be used as a starting material to prepare steric bulk amide **20** with three-stereogenic centers for chiral molecular recognition by ^31^P-NMR analysis (Scheme 16). Furthermore, the linear relationship between the observed and actual ee values of binaphthyl **21** was evaluated and confirmed the accuracy of the ^31^P-NMR analyses. The strategy was based on the method developed by Kagan et al. [59], in which simple chiral amides were studied as CSAs for efficient determination of enantiomers between several types of compounds. The chiral recognition of Kagan’s amide is achieved by hydrogen-bonding using a different procedure than the one outlined by Bedekar et al. The effectiveness of the method was expanded to *alpha*-carboxylic acids by ^19^F-NMR spectroscopy using chiral isobornyl amide **20a** and a base (DMAP) to improve the intermolecular interactions (Scheme 17). In another work of Bedekar et al. a modified Kagan’s amide was synthesized and applied as CSA for hydrogen-bonding based chiral discrimination employing ^1^H- and ^19^F-NMR spectroscopy [60].

**Scheme 18:**
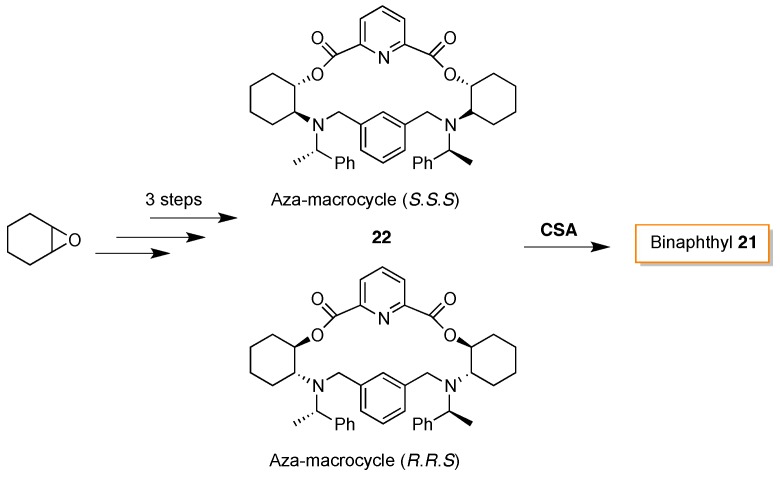
Chiral discrimination of Binaphtyhl **21** using the aza-macrocycle by ^31^P {^1^H} NMR spectroscopy.

The same research group has published the chiral discrimination of binaphthyl **21** by employing a chiral aza-macrocycle **22** [61]. The synthesis of 18 member aza-macrocycles (*S*,*S*,*S*)-**22** and (*R*,*R*,*S*)-**22** was carried out in three steps from the cyclohexene oxide (Scheme 18). To confirm the 3D structure, a single crystal X-ray analysis of both diastereomers was performed. The discrimination ability of aza-macrocycle **22** for binaphthyl phosphoric acid **21** ranged from 0.03 to 0.81 ppm. An additional fluorescence spectroscopy study for understanding the chiral recognition of chiral aza-macrocycle showed an enantioselective quenching interaction. This effect was attributed to the deprotonation of phosphoric acid **21**, which is indicated by the appearance of new peak in the UV-Vis spectra.

**Scheme 19:**
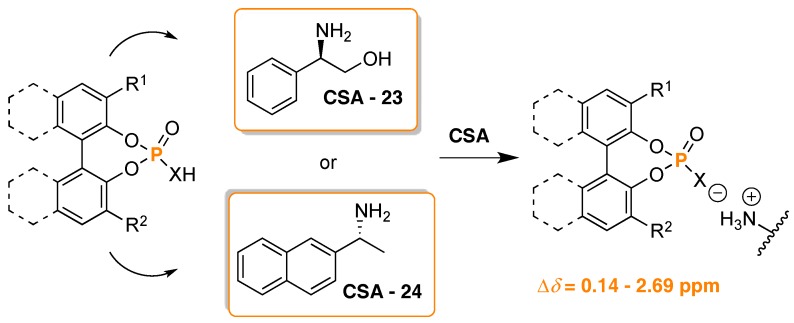
Chiral discrimination of 3,3′-substituted BINOL and H8-BINOL phosphoric acids by ^31^P {^1^H} NMR spectroscopy.

Based on the difficulties in performing the chiral discrimination of BINOL phosphoric acids **21** and derivatives, Nagorny and Tay have developed a simple and reliable protocol for determining the enantiopurity by ^31^P-NMR spectroscopy [62]. The optical rotation measurements were not reliable for these compounds. Thus, the use of chiral amines as CSAs to carry out the NMR chiral discrimination of BINOL phosphoric acids derivatives was an elegant alternative (Scheme 19). Chiral amines **23** and **24** are commercially available and by the simple mixture of reagents (10 mg of BINOL and 1.5 equiv. of CSA) in a deuterated solvent, the ^31^P-NMR signals of the diastereomer salts are observed. The sample concentrations ranged from 4.7 to 25 mM. In this work, the authors studied the epimerization under thermal conditions of the BINOL phosphoric acid derivatives using the ^31^P-NMR protocol developed.

**Scheme 20:**
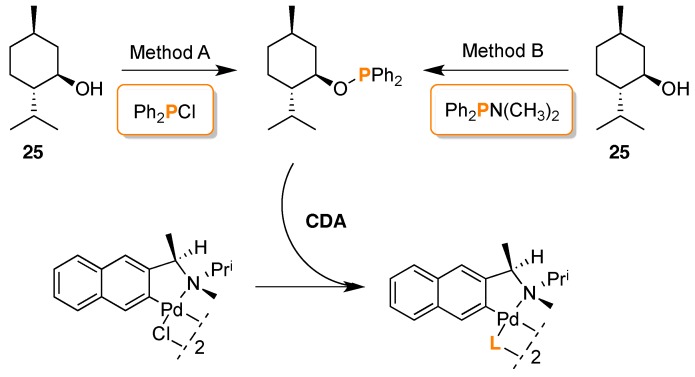
Chiral discrimination of *CN*-palladacycles by ^31^P {^1^H} NMR spectroscopy.

The use of ^31^P-NMR spectroscopy in organometallics chemistry is a well-known tool for structure elucidation and evaluation of mechanisms [63]. Recently, Gorunova et al. have prepared a chiral phosphine ligand as a derivatizing agent for enantiopurity determination of *CN*-palladacycles using ^31^P-NMR [64]. The phosphine ligand was synthesized in one step from the cheap and naturally occurring chiral menthol **25** (Scheme 20). The enantiopurity determination is carried out in situ, without any complications from geometric isomerism or palladacycle dechelation. Continuing this work, the same authors applied this method to determine the absolute configuration of the *CN*-palladacycles [65]. The results were based on ^31^P-NMR chiral discrimination, density function theory (DFT) calculations and X-ray data. The DFT calculations were carried out to study the rotameric mobility of the phosphinite group and dynamiC-NMR spectroscopy and X-ray data were employed to estimate the chirality transfer efficiency in the phosphinite derivatives.

## 4. ^13^C-NMR Chiral Recognition

^13^C-NMR spectroscopy is an indispensable tool for elucidating the structure of organic compounds. Together with ^1^H-NMR analysis, ^13^C-NMR is a routine task in organic synthesis and the study of natural products. The application of this nucleus is hampered by its low abundance relative to ^1^H-, ^19^F-, and ^31^P-NMR, but currently with modern equipment (hardware, magnetic fields, probes, etc.) and new experiments the use of the ^13^C nucleus has become an attractive alternative [66,67,68]. Moreover, the use of ab initio calculations to support the interpretation and assignment of ^13^C-NMR spectra is an important tool for understanding the chiral environment of organic compounds [69].

**Scheme 21:**
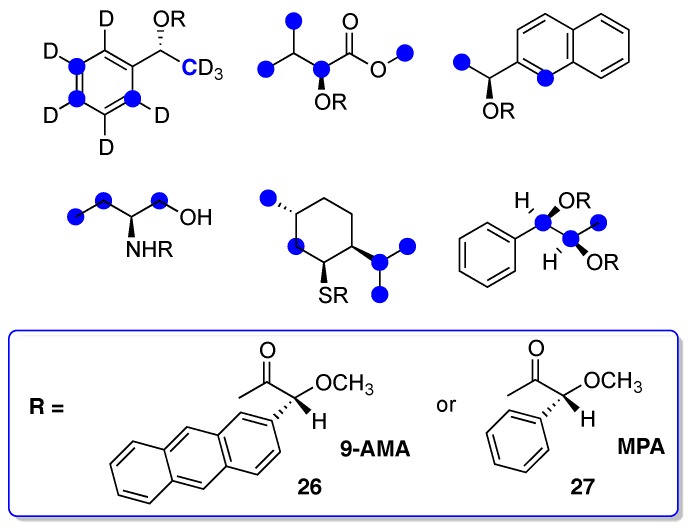
Prediction of Δδ*RS* for ^13^C nucleus of chiral organic compounds.

Riguera et al. have demonstrated the applications and the characteristics that influence the assignment of absolute configuration by ^1^H-NMR spectroscopy [70]. The same research group has recently reported the use of ^13^C-NMR spectroscopy for the assignment of the absolute configuration of alcohols, amines, carboxylic acids, thiols, cyanohydrins, diols, and amino alcohols [71,72]. The authors have examined the ^13^C-NMR data of a collection of chiral samples and the experimental data indicated a perfect correlation between the distribution of signs for ^13^C chemical shifts and ^1^H-NMR of their enantiomers. It is possible to observe that the anisochrony spreads for almost all carbons (Scheme 21: carbons marked in blue; CDAs **26** and **27**). The ^13^C-NMR data follows the same pattern as ^1^H-NMR, providing a way to double check the data. Furthermore, the ^13^C-NMR chemical shifts can be used as a tool for fully deuterated and non-proton-containing organic compounds.

**Scheme 22:**
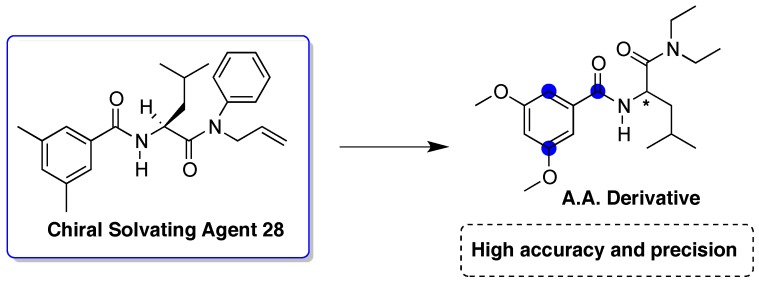
Chiral discrimination of amino acid derivative by ^13^C {^1^H} NMR spectroscopy.

Heo et al. have compared the accuracy and precision of HPLC, ^1^H-, and ^13^C-NMR methods to determine the enantiomeric purity of amino acid derivatives [73]. As shown in Scheme 22, three carbon peaks of (*R*) and (*S*) appeared in different positions. The integration of each peak was automatically performed by NMR software. The total average accuracy calculated from the carbons was 98% and the total average relative standard deviation was 5.23%. The accuracy and the reproducibility of the ^13^C-NMR in comparison to HPLC and ^1^H-NMR were satisfactory for determining the enantiomeric purity of these compounds. The study was carried out by mixing the (*R*) and (*S*) isomers of the amino acid derivative with the CSA **28** in different amounts with a concentration of 20 mM.

For ^13^C {^1^H} NMR, quantification of the chiral discrimination is necessary to prevent significant errors in the NMR analysis. Firstly, the signal/noise ratio should be improved due to the low abundance of ^13^C nuclei (1.1%). Another important parameter is the nuclear Overhauser effect that occurs during the proton decoupling. To reduce this effect, the inverse gated decoupling pulse sequence and the optimized time acquisition must be used. These and other parameters should be evaluated before the NMR analysis to obtain precise multinuclear NMR spectra, mainly for ^13^C nuclei.

**Scheme 23:**
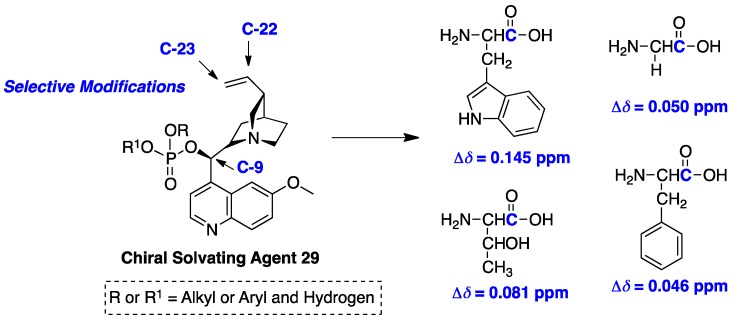
Natural alkaloid **29** employed as a CSA for chiral discrimination of non-derivatized amino acids by ^13^C {^1^H} NMR spectroscopy.

The development of new chiral auxiliaries to determine the enantiomeric excess or to assign the absolute configuration by NMR spectroscopy is a difficult and time-consuming task. For this reason, the use of natural products as a scaffold, either directly or as a starting material to prepare new chiral agents, is an elegant alternative. Based on this strategy, Szostak et al. have employed naturally occurring *Chinchona* **29** alkaloid and their derivatives as CSAs for different functionalities [74]. Selective modifications of the natural products were performed by tuning their enantiodiscrimination. The structure of quinine enables modulation, mainly for carbons *9*, *22*, and *23* (Scheme 23). Moreover, the presence of a phosphate group supplementing the quinine frame core provides zwitterionic character because it possesses two charged functions, a quaternary ammonium cation and a phosphate anion. These characteristics were used with success for enantiodiscrimination of non-derivatized amino acids (Scheme 23). The charges increase the interaction potency between the molecules, allowing for efficient chiral discrimination in the DMSO-*d*_6_ solvent by ^13^C-NMR spectroscopy. The split signals of ^13^C-NMR ranged from 0.025 to 0.145 ppm.

**Scheme 24:**
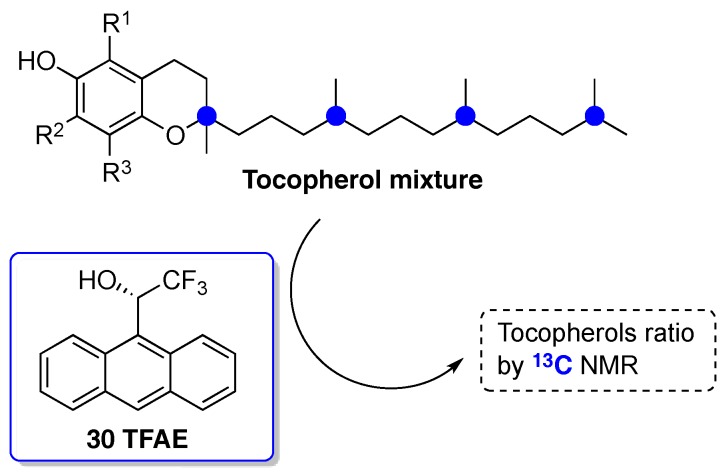
Discrimination of tocopherols stereoisomers using the CSA **30** (TFAE) by ^13^C {^1^H} NMR spectroscopy.

^13^C {^1^H} NMR spectroscopy is also a useful tool for discrimination and analysis of complex mixtures because of the valuable information contained in the carbon spectrum. This strategy becomes more attractive mainly when the ^1^H-NMR spectra demonstrate broad multiplets and small chemical shift differences between the stereoisomers. As a result, Lankhorst et al. presented a simple ^13^C-NMR methodology for the discrimination of a complex mixture of α-tocopherol stereoisomers [75]. The traditional methods employed to perform this discrimination, such as gas and liquid chromatography, require multiple steps to prepare the sample and obtain the ideal conditions. In contrast to the ^13^C-NMR method, the tocopherol mixture and the chiral trifluoroethanol (TFAE) **30** are dissolved in CDCl_3_ to prepare the sample for NMR stereodiscrimination analysis (Scheme 24). Furthermore, the assignment of individual stereoisomers was possible by the synthesis of each tocopherol stereoisomer from stereochemically pure or enriched building blocks. In this work, the temperature-dependent behavior of the racemate in the presence of TFAE **30** was studied. The temperature ranges affected the efficiency of the ^13^C chemical shift differences.

**Scheme 25:**
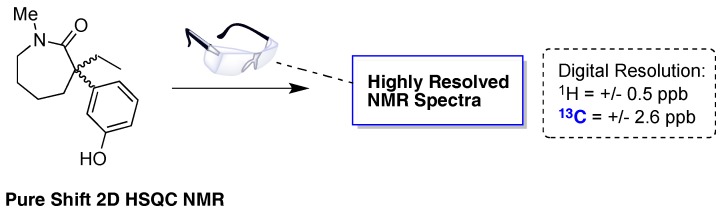
Highly resolved pure shift HSQC-NMR spectra of lactam.

Another route to improve the chiral recognition methodologies by NMR spectroscopy is the development of new pulse sequences. In this sense, a significant increase in the number of new NMR experiments has occurred in the last decades. Among these new experiments, “pure shift” NMR emerged as a useful tool for chiral studies. This pulse sequence and their derivatives have improved the resolution of 1D and 2D NMR experiments to simplify the typical *J*_(H,H)_ multiplicity pattern of ^1^H signals to singlets. The Se-NMR experiments make the NMR stereodiscrimination methods simpler and more efficient, mainly for complex and overcrowded resonances. Recently, Parella et al. have exploited the application of highly resolved pure shift heteronuclear single quantum coherence (HSQC) spectra for ^1^H- and ^13^C-NMR enantiodifferentiation [76]. In this study, the authors employed a racemic mixture of lactams with (*R*)-PA as a CSA to show how the highly resolved 2D HSQC spectra can be used to detect and accurately quantify very small anisochrony values (Scheme 25). The values obtained from the pure shift HSQC were compared to traditional HSQC and 1D NMR experiments, confirming the advantages of this new pulse sequence.

Stereochemical NMR studies have also received attention based on the development and application of chiral oriented media, such as liquid–crystalline systems [77] and stretched polymer gels [78]. The main use of these oriented media is for ^2^H-NMR experiments due to the quadrupolar moment of the deuterium nucleus. Chiral oriented media is a flexible approach that can be applied to several organic solvents without the use of solid-state NMR [79]. For this approach, a chiral liquid–crystalline is put into an NMR tube and after addition of an organic solvent and the probe the chiral liquid crystal swells and forms a gel. The gel does not swell isotropically but anisotropically along the glass wall of the tube and the resulting align–angular information relative to the static magnetic field can be obtained. This method is already a common procedure used for biomacromolecules in aqueous solution.

Based on these features, the chiral recognition by liquid–crystalline systems was extended to other nuclei. The use of ^13^C-NMR spectroscopy is a valuable alternative to discriminate chiral environments in oriented media due to a much larger dispersion of chemical shifts. Furthermore, the correlation between 2D NMR experiments for ^13^C and ^2^H isotopes has the benefit of different pulse sequence as the INEPT-DECANCY and DEPT-DECANCY to improve the resolution and sensitivity. These characteristics were well described by Lesot et al. in a recent review [80]. The use of ^19^F-NMR spectroscopy for the measurement of enantiomeric excess was successfully extended to oriented media by Phillips and Sharman [81].

## 5. ^77^Se-NMR Chiral Recognition

Selenium organic compounds are related in a broader context of applications [82,83,84] and selenium is a fundamental component of the living cells of a variety of organisms [85]. Based on these features, the use of ^77^Se-NMR spectroscopy emerged as an opportunity for structure and reactivity studies [86].

**Scheme 26:**
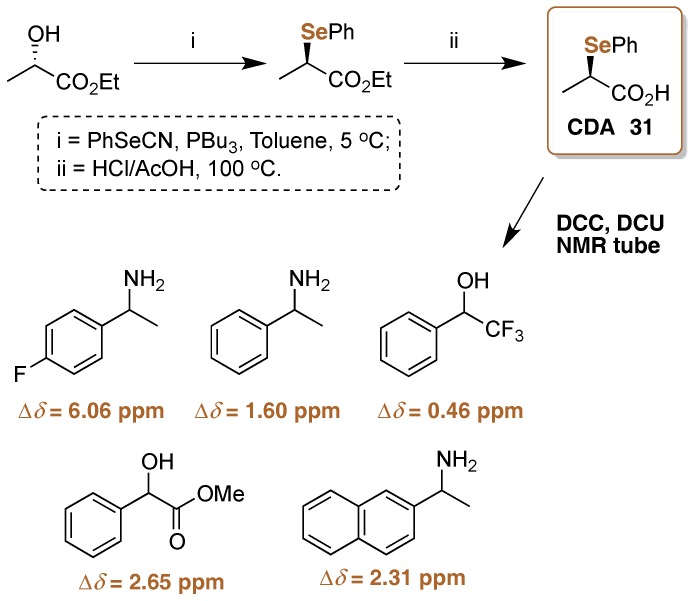
Chiral discrimination of alcohols and amines employing the selenide CDA **32** as a probe by ^77^Se {^1^H} NMR spectroscopy.

The application of ^77^Se-NMR spectroscopy for chiral recognition has great potential for success due to the characteristics of the selenium nucleus, such as a wide spectral window, reasonably high natural abundance, especially compared with the carbon nucleus, and because the ^77^Se isotope is ½ and shows no significant nuclear Overhauser effect. Thus, the possibility of developing chiral probes containing selenium has several benefits. In this context, Orlov and Ananikov synthesized a chiral selenide probe as a CDA for determination of enantiomeric purity of alcohols and amines by ^77^Se-NMR spectroscopy [87]. The synthesis was performed in two steps achieving 80% yields of CDA **31** (Scheme 26). Selenide chiral probe **31** was successfully applied to enantiodiscrimination of a variety of alcohols and amines.

**Scheme 27:**
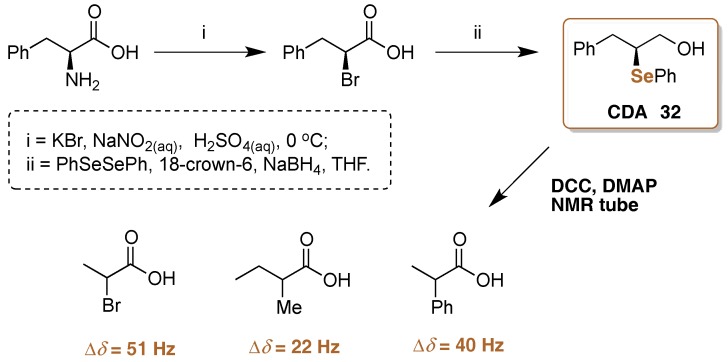
Chiral discrimination of carboxylic acids employing the selenide CDA 33 as a probe by ^77^Se {^1^H} NMR spectroscopy.

Ferreira and Gonçalves have carried out an inverse planning procedure to prepare a new chiral selenide alcohol as a CDA [88]. The article describes the synthesis of chiral selenide alcohol **32** in two steps (Scheme 27). To perform the derivatization, a ”*mix and shape*” method was performed in an NMR tube, similar to Orlov and Ananikov’s work. Using selenide alcohol **32**, chiral discrimination was achieved for different carboxylic acids.

**Scheme 28:**
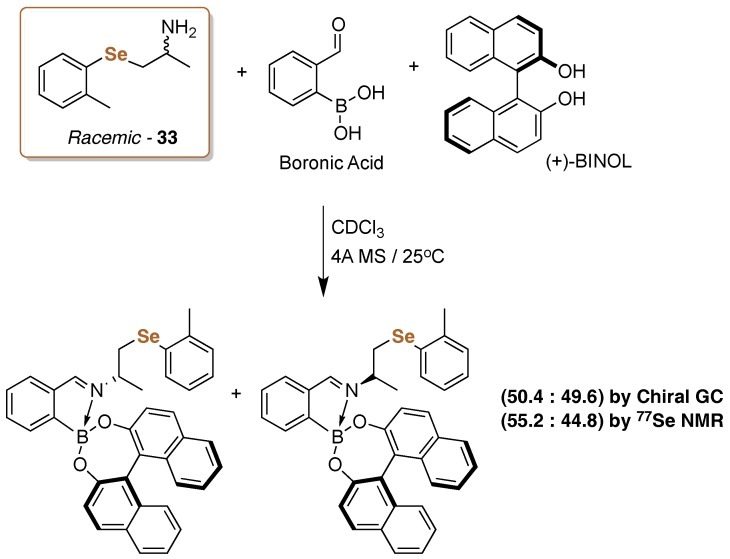
Three-component reaction for chiral discrimination of selenide amines by ^77^Se {^1^H} NMR spectroscopy.

Recently, Silva et al. have demonstrated the application of a three-component reaction for chiral discrimination of amines **33** containing the selenium atom by ^77^Se-NMR spectroscopy [89]. This simple and inexpensive chiral derivatizing method was successfully employed for a variety of organic compounds by ^77^Se-NMR. In this protocol, the selenide amine reacts with 2-formylphenylboronic acid and the active (+)-BINOL in the presence of molecular sieves for 10 min (Scheme 28). The ^1^H-NMR analyses were dependent on the concentration. In highly concentrated media the signals were broader. It is possible that a self-aggregation interaction occurred during the analysis. For ^77^Se-NMR spectra, this concentration effect was not observed. Moreover, an HPLC analysis was performed for comparative evaluation of the ^77^Se-NMR results (Scheme 28).

**Scheme 29:**
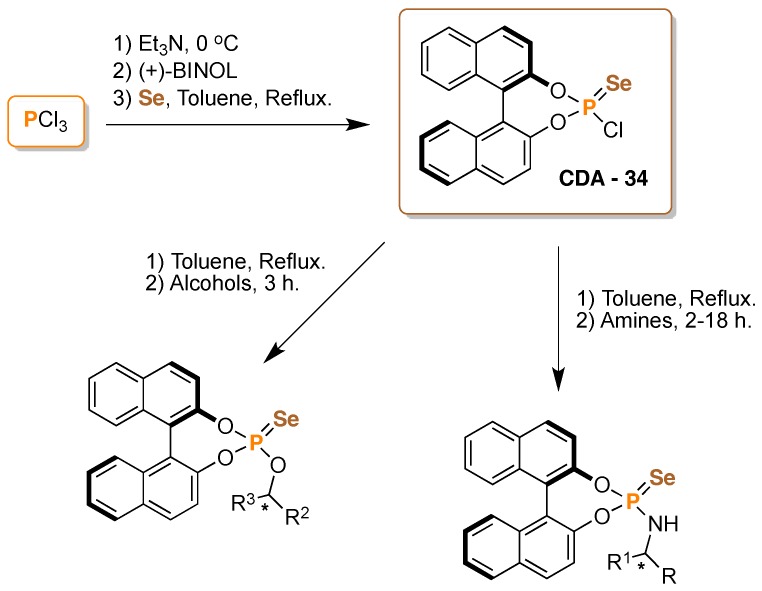
Phosphoroselenoic acid derivatives for chiral discrimination of amines and alcohols by ^77^Se {^1^H} and ^31^P {^1^H} NMR spectroscopy.

Murai has performed the synthesis of the phosphoroselenoic acid **34** as a double check chiral discrimination protocol for amines and alcohols by ^77^Se- and ^31^P-NMR spectroscopy [90]. The CDA **34** was synthesized in a one-pot procedure achieving 94% yields (Scheme 29). All CDAs obtained are stable under air and are purified by column chromatography. The absolute configuration of the CDA **34** was confirmed by X-ray analysis. The CDA **34** showed high reactivity for primary amines and alcohols. When tertiary alcohols were employed the reaction did not proceed. Moreover, most of the diastereoisomers formed by this protocol were separated by simple recrystallization. Recently, Murai and Itoh have applied these CDAs derivatives for the ^77^Se- and ^31^P-NMR discrimination of remote chirality of primary alcohols [91].

## 6. Conclusions

Numerous efficient and versatile chiral derivatizing and solvating agents have emerged in recent years. These compounds present different chiral recognition abilities since the structure and conditions can be tuned to improve the molecular interactions. With the expectation of expanding the chiral probes, using multinuclear NMR spectroscopy provides new possibilities for chiral studies and the elucidation of their chiral recognition mechanisms. ^19^F, ^31^P, ^13^C, and ^77^Se nuclei have demonstrated efficient results based on their nuclear properties and their incorporation into organic compounds through organic synthesis procedures.

Although the use of multinuclear NMR spectroscopy for chiral discrimination and assignment of the absolute configuration is attractive, we need to be aware that the reliability of the multinuclear NMR analysis should be evaluated for the respective application due to the different nuclear properties and general behavior of each element.

In order to spread the use of multinuclear NMR, new pulse sequence and experimental conditions were developed to improve the time-consuming and accuracy of the NMR spectra. Furthermore, the information obtained from these new experimental procedures can be employed to complement the traditional results and/or used for specific purposes.
